# Truly Target-Focused Pharmacophore Modeling: A Novel Tool for Mapping Intermolecular Surfaces

**DOI:** 10.3390/molecules23081959

**Published:** 2018-08-06

**Authors:** Jérémie Mortier, Pratik Dhakal, Andrea Volkamer

**Affiliations:** In-Silico Toxicology Group, Institute of Physiology, Charité—Universitätsmedizin Berlin, Virchowweg 6, 10117 Berlin, Germany; jeremie.mortier@gmail.com (J.M.); dhakal.pratik@gmail.com (P.D.)

**Keywords:** target-focused pharmacophore modeling, density-based clustering, structure-based drug design, AutoGrid, grid maps, probe energies, method development

## Abstract

Pharmacophore models are an accurate and minimal tridimensional abstraction of intermolecular interactions between chemical structures, usually derived from a group of molecules or from a ligand-target complex. Only a limited amount of solutions exists to model comprehensive pharmacophores using the information of a particular target structure without knowledge of any binding ligand. In this work, an automated and customable tool for truly target-focused (T²F) pharmacophore modeling is introduced. Key molecular interaction fields of a macromolecular structure are calculated using the AutoGRID energy functions. The most relevant points are selected by a newly developed filtering cascade and clustered to pharmacophore features with a density-based algorithm. Using five different protein classes, the ability of this method to identify essential pharmacophore features was compared to structure-based pharmacophores derived from ligand-target interactions. This method represents an extremely valuable instrument for drug design in a situation of scarce ligand information available, but also in the case of underexplored therapeutic targets, as well as to investigate protein allosteric pockets and protein-protein interactions.

## 1. Introduction

Events of intermolecular recognition are mediated through forces of attraction and repulsion between interacting chemical molecules. From the transduction of extracellular signals to DNA recognition by transcription factors, all combinations of interaction partners result in unique molecular complexes with a particular biological significance for a given cellular time and space. Therefore, studying the specificity of particular interactions at the atomic level is essential for comprehending biochemical mechanisms and predicting molecular behaviors. Depicting the ensemble of key interactions required for a specific intermolecular recognition event is the working principle of the pharmacophore approach. A pharmacophore model is made of a set of interactions, typically consisting of hydrogen bonds, electrostatic interactions, and π-stacking, as well as hydrophobic contacts, and may also include steric information, such as exclusion volumes. Recording the 3D-arrangement of all interaction features included in a pharmacophore model has been highly facilitated with the advent of computational chemistry [1].

Pharmacophore modeling is a computationally efficient and pragmatic strategy for the discovery and optimization of biologically active compounds [2,3,4,5], as well as the analysis of intermolecular interactions in silico [6,7]. Because of their simplicity, these intuitively understandable models can support binding event prediction for a selected group of molecules or to conduct high-throughput virtual screening of large compound libraries [8]. For a set of molecules sharing a similar biological response, a ligand-based pharmacophore model can be derived by superposing them and determining the maximum number of overlapping chemical features [2]. This method is called ligand-based approach. When structural information is available for an intermolecular surface involving multiple partners, a pharmacophore model can be derived from the interactions detected in this complex. This method is called structure-based approach, and is typically conducted by geometrically deriving features from a ligand-target complex [9]. While the label, structure-based approach, primarily evokes an investigation focused on a protein’s structure, only a limited amount of solutions exists to model comprehensive pharmacophores using information of a particular target structure without considering the binding mode of a ligand [10]. The consequences of this situation are that (i) a restricted number of options are available to build pharmacophore models for targets when no binding ligand is known, and (ii) when a small amount of ligands that bind to a target are known, the resulting structure-based pharmacophores are limited to the interactions these particular molecules are forming, poorly representing the range of pharmacomodulations available for rational drug design (or pharmacophoric space). With the rising number of protein structures available, target-focused techniques will play an increasingly important role to elucidate biological functions and to support target-oriented drug design. Nowadays, protein structures are solved sometimes before anything is known about their biological function, e.g., by proteomics approaches. It appears clearly then that reliable methods capable of deriving pharmacophore models from ligand-free protein structures (and any other relevant biomolecular surfaces) are needed.

Target-focused pharmacophore models can be derived based on evolutionary pocket residue conservation (alignment), by minimizing probes (molecular dynamics), or by identifying favorable energetic properties (grid-based). The former two will only be shortly covered, while the latter one is the focus of this work.

Sequence alignment-based methods rely on the detection of key residues of a binding site for subsequent pharmacophore features assignment [11,12]. As an example, this approach was followed and developed for the construction of a G-protein coupled receptor (GPCR) pharmacophore model [13]. In this work, Kratochwil et al. aligned over 1000 sequences of GPCRs to identify patterns of residues conserved in the transmembrane domain across this protein family. Once aligned to the target structure, important positions for ligand binding and key residues for selectivity were selected to assign pharmacophore features. Clearly, this strategy can be effective in a scenario with sufficient data available, such as in the case of GPCRs, but has little chance of success with a less explored protein class.

An alternative approach is to simulate the dynamic behavior of chemical probes (water and organic solvents) on a flexible molecular surface [14,15]. The minimized probe molecules can unveil favorable interaction sites on the protein surface, which can be converted into pharmacophore features. A first attempt by Miranker and Karplus [14,15] in 1991 to include protein flexibility in a pharmacophore model led to a number of promising developments of computer-based investigations of macromolecules using molecular dynamics (MD). Although more time consuming, this approach can be successful when water, the natural solvent of proteins, is used as a probe. However, organic probes used to detect the hydrophobic regions of a macromolecule create a non-natural environment for most proteins, inducing conformations in silico that are unlikely to be observed in vivo. The relevance of the resulting pharmacophore model is therefore unclear. Current challenges and perspectives of this approach are discussed in this recent review [16].

To date, the most established method to derive pharmacophore models from an empty molecular surface or cavity is to identify regions with the most favorable energetic properties for ligand binding. In 1985, Goodford developed an efficient and straightforward method for sampling protein cavities [17]. This method is based on the determination of the chemical nature of a molecular surface by calculating interaction energies in the presence of chemical probes with different electronic properties, such as hydrogen bond donating or accepting groups, as well as hydrophobic ones. A three-dimensional (3D) Cartesian grid box subjected to energy calculation is spanned around the area of interest [17,18], and grid points with the most favorable energetics identify regions of attraction between a potential ligand and the studied macromolecule. Among the known grid-based approaches developed for scanning a target surface to identify hot spots (Table 1), *Pocket V2* [19], *GBPM* [20], Tintori et al. [21], *Hydro-Pharm* [22], *FLAP* [23], and *PharmDock* [24] opted for a solely energy-based 3D grid approach, while *Ph4Dock* [18] builds upon a sphere-based cavity detection method. Some additional methods are available in modeling suites, such as the geometry-based GridMap approach in LigandScout, to derive a pharmacophore model from an empty cavity, but the methodology has not been published and therefore cannot be discussed in detail in this overview. Most of these methods were developed for docking applications. Ph4Dock [24] compares the derived protein pharmacophores to those of ligands to rank docking conformations. *FLAP* [23] converts the derived molecular interaction fields into fingerprints and is applied for high-throughput virtual screening. Hydro-Pharm [22] and PharmDock [24] use ChemScore-based energies [25] calculated on grid points to identify pharmacophore features for subsequent molecular docking. In contrast, PocketV2 [19], GBPM [20] (focused on protein-protein interactions), and the method by Titori et al. [21] were primarily developed for pharmacophore detection, all using an energy grid for hot spot detection based on point clustering (former) or minima extraction (latter two).

*Pocket definition:* For applying target-based methods to rather unexplored—ideally, to apo—structures, the location of the (potential) ligand binding site needs to be determined. However, some methods, such as *Hydro-Pharm* [22] *or PharmDock* [24], require a ligand to define the cavity volume (centered on the ligand coordinates). Some others, such as *Pocket V2*, additionally allow the user to input selected binding site residues to define the pocket. In contrast, *Ph4Dock* [18] is a ligand docking program that provides an inbuilt pocket detection routine by scanning the surface with a collection of spheres using a modified Delaunay triangulation [35]. *BioGPS* [31], which builds upon *FLAP* [23], includes the *FLAPsite* algorithm for pocket detection, using the GRID H-probe for sampling the complete protein surface together with a distance and buriedness filter, combined with an erosion (removing small anomalies) and dilation (filling holes) procedure. An alternative to an inbuilt pocket detection step would be the use of external binding site detection methods. Several such approaches exist, e.g., *SiteMap* [36], *Fpocket* [37], or *DoGSiteScorer* [38], that use geometric or energetic features of the protein surface to identify points in protrusions and cluster them to cavities [39]. Note that, in this case, the binding site detection method needs to return e.g., the center of the cavity and the size, and the pharmacophore method must be able to process this input.

*Filter & clustering procedures:* Once the binding site is defined, energy levels can be calculated using different probes sampled along the 3D grid and the most favorable regions for binding can be identified and translated into pharmacophore features. For each interaction type, grid points with the best energetics must be assembled accurately to derive the corresponding pharmacophore feature. To achieve this non-trivial task, a clustering method must be implemented, and the choice of the clustering algorithm will have a major impact on the resulting model. *Ph4Dock* [18] opted for the single-linkage clustering algorithm, a hierarchical clustering method grouping clusters from bottom to top (agglomerative clustering). This method allows fast processing of large data sets by grouping points separated by the smallest distances. As a consequence, the single-linkage method creates long clusters in which two points at the opposite ends of the same cluster can be more distant to each other than other points from neighboring clusters [40]. K-means clustering, used in *Hydro-Pharm* [22] and *PharmDock* [24], belongs to the class of partitioning cluster algorithms. The approach of k-means is to divide the dataset into *k* clusters. The algorithm starts by randomly selecting *k* seeds (cluster centroids) and annotates the points to the closest centroid. Iteratively, the cluster centroids are recalculated and the points are annotated to the updated centroids. This is repeated until the assignments remain constant and the system converges. A disadvantage of the k-means algorithm is that it requires the user to specify the amount of clusters, *k*, as an input. When the dataset is a group of points distributed on a 3D grid, defining a strict number of clusters significantly affects the resulting pharmacophore model, e.g., by placing the center of a cluster between two hot spots if they end up in the same cluster. A third clustering approach, named common nearest neighbor (CNN) algorithm and developed by Keller et al. [41], reproduces what human intelligence intuitively distinguishes as clusters in a group of points [42]. In contrast to most geometric cluster algorithms, which are founded on the notion that members of a cluster are closer to each other than to all other points in the data set (such as k-means), the cluster definition in this approach is based on a measure for the local data point density. The CNN algorithm displayed the best aptitude to cluster five two-dimensional test cases in a comparison that included a simple hierarchical clustering algorithm (related to single-linkage) and a partitional algorithm (related to k-means) [42,43]. Initially developed and tested for clustering molecular dynamic trajectories [34,42], the CNN algorithm can be applied to any set of 3D grid points and has, to the best of our knowledge, not yet been used for pharmacophore perception.

*Application & novelty:* Since methods, like *Ph4Dock* [18] or *PharmDock* [24], were primarily developed for improving docking algorithm performances or for efficient high-throughput virtual screening (*FLAP* [44]), these methods mostly calculate a high amount of pharmacophore features in the cavity to improve scoring. However, deriving a large number of features is not suitable for the representation of a pharmacophoric space in a simple, straightforward, and readable manner for a medicinal chemist (typically, structure-based and ligand-based models are made of three to eight pharmacophore features). For the obvious reason of efficiency, a reduced amount of features is also required for improving the calculation time in the frame of large library screening. However, most importantly, an ability to detect the most critical interactions for optimal binding clearly emerges as a key feature for the development of a new target-focused method. Even though pharmacophore modeling is historically bound to medicinal chemistry, the spectrum of applications related with the study of intermolecular recognitions is broader than simply the docking of potential new drugs. All researchers investigating multivalent systems can benefit from a fast and reliable method for molecular surface analysis and target-focused pharmacophore modeling. As it appears from this literature overview, a simple and robust method to automatically derive reliable pharmacophore models from empty macromolecule structures is urgently needed. In this paper, we report the development and evaluation of a novel computational tool combining the AutoGRID energy function, an advanced cavity annotation, and the CNN clustering algorithm for the design of truly target-focused pharmacophore (T^2^F-Pharm) models.

## 2. Materials and Methods

### 2.1. Pharmacophore Generation

**Figure 1:**
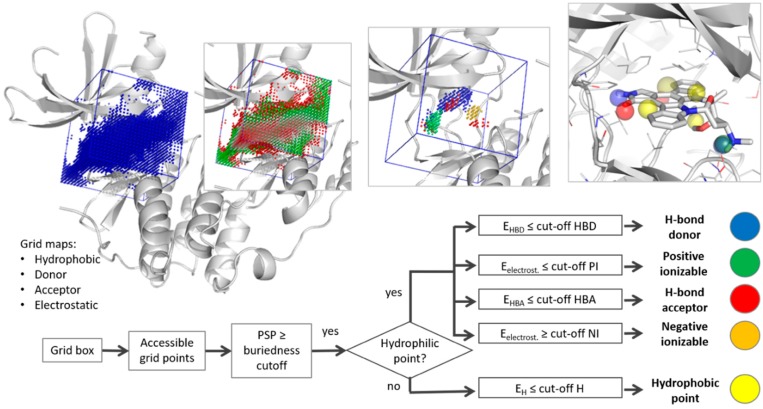
Graphical representation of the main steps of the decision tree invoked by the truly target-focused (T^2^F) pharmacophore method.

The algorithm developed in the T^2^F approach relies on the following steps, as illustrated in Figure 1.

*Grid box:* In the first step of the T^2^F method, the domain of interest for the hot spot calculation is defined, with or without a co-crystallized ligand. If a ligand is present in the crystal structure, the center of mass of all ligand heavy atoms is automatically calculated and chosen as the grid center. If no ligand information is given, the grid center coordinates can be defined manually. In this case, either user knowledge about the location of the active site is necessary or a pocket detection method, such as DoGSiteScorer [38] (freely available on the ProteinsPlus server [45]), can be evoked. The size of the 3D grid is determined by the edge length of a cubic volume, and the density of the grid by the space between two points. A box edge size of 16 Å and a grid spacing of 0.6 Å are used by default.

*Energy calculation:* To sample the macromolecular surface of interest, a 3D grid is spanned around the specified targeted surface [46]. The freely available AutoGrid functionality within AutoDock [33] is then used to sample the energies on the grid points. Energy grid maps are calculated by positioning four different probes on each point of the 3D grid to determine the chemical nature of the macromolecular surface. The chosen probes, included in the AutoDock package [46], are (i) an aliphatic carbon for hydrophobic contacts (H-probe), (ii) a hydrogen that donates a hydrogen bond (HBD-probe), (iii) an oxygen accepting hydrogen bonds (HBA-probe), and (iv) a charged group to calculate electrostatic interactions (i.e., to describe positive (PI) and negative ionizable (NI) groups).

*Cavity points filtering:* Accessible points are distinguished from those occupied by protein atoms using the energy values obtained from the hydrophilic hydrogen acceptor probe. Sterical clashes with the protein surface result in high energy values. Thus, grid points with an energy value below a given cut-off are considered as accessible, and as occupied otherwise. To further discard points that are too far from the surface, and thus outside of the cavity boundary, the buriedness of each grid point is calculated. Therefore, so called *protein-solvent-protein* (PSP) events, as described by Hendlich et al. [47], are determined. Each grid point is scanned in seven directions (x-, y-, z-axis, and the four diagonals) and the number of PSP events per grid point is calculated. Only grid points above a certain PSP buriedness cut-off are kept for the pharmacophore perception step. Per default, a PSP value of 4 is used.

*Pharmacophore perception:* To assign pharmacophore features, the remaining (accessible and buried) grid points are filtered and grouped based on their interaction type. First, hydrophilic and hydrophobic points are separated. To determine whether the region around a point is hydrophilic, its surrounding protein residues within a defined radius are scanned for hydrophilic atom types. PyMol [48] is used to span a sphere around each grid point and to collect all amino acid atoms inside this sphere. Points that are surrounded by at least one atom with the potential to function as a hydrogen bond donor or acceptor are considered hydrophilic. The assignment as a potential donor or acceptor is based on the annotation of the respective function encoded in the atom names in the PDB file using the IUPAC-IUB rules. If no hydrophilic atom is detected in this environment, the points are considered hydrophobic. Second, after separating the hydrophilic from the hydrophobic points, they are filtered by energy level using a type-specific cut-off. This minimum energy value is different for each probe. All points with an energy level above the cut-off are discarded. Third, the remaining points are clustered into low energy hot spots for each interaction type using the CNN clustering algorithm [41,42]. To sort low energy hot spots into clusters, two parameters are needed: Distance and similarity cut-offs. The distance cut-off defines the area of a neighborhood for each point, and the similarity cut-off assigns the minimum number of neighbors that two points need to have in common to belong to the same cluster [42]. Larger clusters (more than 80 grid points) are re-clustered with a higher similarity value to be fractioned into multiple moderate sized clusters. This additional step is useful to better describe two neighboring hotspots of the same interaction type, which would otherwise be represented as one dense cluster (often observed for hydrophobic pockets). Subsequently, clusters with less relevance, i.e., those including less than 15 points, are discarded. Finally, a T^2^F pharmacophore model is derived by assigning the center of each remaining cluster as one pharmacophore feature. Importantly, the volume of a feature is relative to the amount of points included in the cluster. For this, a sigmoid function variation is used where the feature size responds to the cluster volume change within a radius range of 0.75–2.75 Å.

*Pharmacophore processing:* Output files generated by the T^2^F software are readable (i) in PyMol for visual analysis (pseudo pdb and cgo files), and (ii) in LigandScout (pml file) for further pharmacophore alignment, evaluation, and subsequent virtual screening, as well as (iii) in LeadIT (phm files) for pharmacophore-based docking.

### 2.2. Evaluation Data Sets

*Target data set*: The Patel set [29] contains a collection of five well-described protein families, for which a large amount of structural data is available and has been used to evaluate other tools in the context of pharmacophore modelling. In our set, four groups of structurally diverse proteins were collected (the zinc-containing protein, thermolysin, had to be discarded in this first evaluation as this version of the T²F method is not suitable for the detection of metal coordination). The set contains ligand-enzyme complexes for (a) cyclin-dependent kinase 2 (CDK2, 6 entries), (b) dihydrofolate reductase (DHFR, 6 entries), (c) thrombin (7 entries), and (d) HIV-reverse transcriptase (RT, 10 entries). In addition, the adenosine A_2A_ receptor was included in our set (3 entries) to represent the GPCR class of proteins. The target data set is summarized in Table 2.

*Structure preparation:* All structures were downloaded from the Protein Databank (PDB) [49] and ProToss (included in the *ProteinsPlus* server [45]) was used to calculate optimal hydrogen bonding networks. All structures were pre-aligned per protein group to allow comparison of pharmacophores derived from different structures (using the *super* function in PyMOL [48]). If a cofactor or water molecule is important for ligand binding, it can be conserved in the protein file.

*Experiment set-up:* A T^2^F pharmacophore model was generated for one representative ligand-protein complex of the five protein families of the target set after removing the co-crystallized ligand. The name and detailed information of the selected reference structure are reported in Table 2.

In order to compare T^2^F pharmacophores to structure-based (SB) models, LigandScout [50] was used to derive an SB pharmacophore from each ligand-protein complex of the target set [29]. Except for thrombin (PDB entry 1C4V), water molecules were removed, SB pharmacophore models were built using default parameters, and exported in a pml format for subsequent analysis. If known key interactions were not detected with the default parameters, the small molecule was minimized using MMFF94 [26] and a new SB pharmacophore was built. If this key interaction was detected then the model with the minimized molecule was kept. Otherwise, the SB model derived from the non-minimized conformation was chosen.

*Match calculation:* A match between a T^2^F and an SB pharmacophore feature is reported if two features of the same type are found within a maximum distance of 2 Å from one another. For T^2^F features, the center of the feature is taken as the reference point for all types. For SB features, the same holds true for the hydrophobic features (represented as spheres), whereas, for H-bond features (represented by arrows in LigandScout), the position of the H-bond donating or accepting heavy atom is taken as the reference. Note that the HBD feature location is different to the T^2^F method, which samples favorable positions for the H-bond donating H-atom. This difference results in a slight feature shift observed in the following evaluation. Finally, the root means square deviation (RMSD) between all matching features is calculated for each pair of T^2^F—SB pharmacophore models.

## 3. Results and Discussion

In the following section, the selected parameters will be discussed, then the generated T^2^F-Pharm models for the five proteins of our target dataset will be presented and analyzed.

### 3.1. Parameter Selection

*Grid box:* The typical volume of a druggable cavity is around 900 Å^3^ [51,52]. To cover the complete volume of typical drug binding sites (including larger ligands) with a cubic box, the grid is spanned over a box with a 16 Å edge length. For grid spacing, values between 0.4 Å [32] and 1.0 Å [44] have been reported in the literature for grid representations of cavities. While the usage of smaller grid spacing can compensate for discretization effects, it comes with an exponential increase in grid points and, thus, in calculation time. A grid delta (space between two grid points) of 0.6 Å was an ideal compromise between accuracy and efficiency.

*Cavity points*: To determine the actual ligand accessible pocket volume in the grid box, first, points clashing with the protein are rejected. Then, points that are too far from the protein surface are filtered out. Since energies are calculated on each grid point, those occupied by protein atoms will receive unfavorable energy values independently of the probe type. In this work, the energy of the hydrogen bond acceptor probe (HBA) was chosen for discriminating free from occupied grid points, using an energy cut-off of 0.6 kcal/mol as a good compromise of excluding points in small voids while keeping those close to the protein surface. The chosen occupancy value reduces the grid points to be considered to, on average, 30% of the total amount of grid points (about 20.000 points in a box) in the target set discussed hereafter. To exclude points distant from the protein surface and outside the typical interaction radius of a ligand, a buriedness filter is applied. PSP values calculated as described in LIGSITE [47] range from 0 (the most solvent-exposed) to 7 (the most buried). A PSP value ≥ 4 delivered meaningful results in previous pocket-related work [38] and was therefore chosen for this study. This buriedness cut-off further restricts the number of considered grid points to, on average, 14% of all grid points in the original grid box.

*Hydrophilic points*: To separate hydrophilic from hydrophobic points, the surrounding protein atoms of each grid point are scanned. A point is considered hydrophilic if at least one hydrogen-bond donating or accepting protein heavy atom is detected within a radius of 3 Å. This value corresponds to the typical distance of 2.8–3.2 Å found in protein-ligand complexes between two heavy atoms forming a hydrogen bond [53]. As the distance increases, hydrophilic interactions tend to become weaker and, hence, less reliable (note that the optimal angle for forming a hydrogen bond is not taken into consideration given that the probe is a single atom).

**Figure 2:**
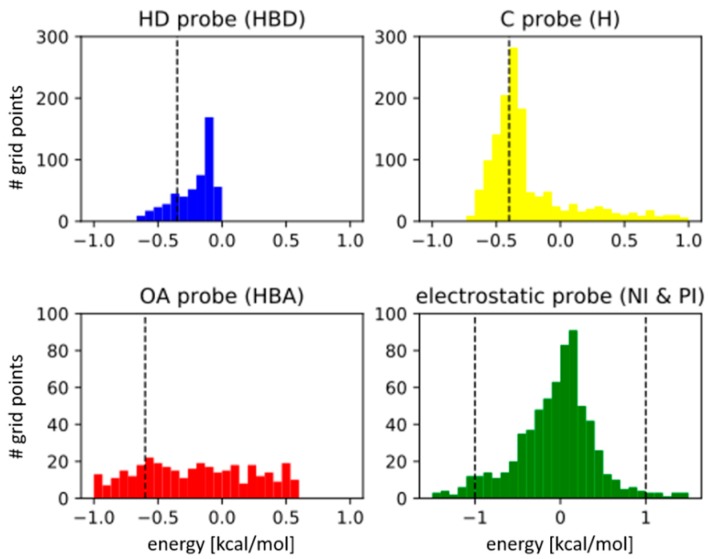
Energy distribution of the free and buried cavity points from the cavity of cyclin-dependent kinase 2 (CDK2) (Protein Databank (PDB) entry 1AQ1) for the four different probes: H-bond donor (HBD in blue), hydrophobic (H in yellow), H-bond acceptor (HBA in red), and electrostatics (in green). The respective energy cut-offs are represented by a dotted vertical line (points with an energy value above the cut-off are discarded). Note that for electrostatics both extrema are kept, describing positive and negative ionizable (PI and NI) areas, respectively.

*Energy cut-offs per probe:* Default parameters per grid probe were selected based on an analysis of the individual probes energy value distributions. The cut-offs were chosen in a way that only the most energetically favorable grid points are retained for each interaction type, as illustrated by the energy distributions in the four histograms shown in Figure 2. Points with a calculated energy below the individual cut-offs are selected for the clustering procedure. A summary of all energy cut-offs can be found in Table 3. While the default energy cut-offs delivered good results for most of the reported cases, the active site of HIV-reverse transcriptase (PDB entry 1TVR) was found to be highly hydrophobic. Thus, cut-offs were adapted to avoid large hydrophobic clusters and to derive the most meaningful pharmacophore features.

*CNN clustering:* The CNN clustering algorithm is a novel local density-based method [34] and the script was kindly shared by their authors for implementation in the T^2^F method [41]. For the neighbor distance cut-off, a value was chosen that spans a sphere with a radius of 1.21 Å around each point, thus, including the two surrounding shells of points on a 0.6 Å-spaced grid. Two points need to have at least six neighboring points in common to be allocated to the same cluster. Since hydrophobic areas tend to be more bulky, we introduced a hierarchical re-clustering if the resulting clusters are too large (>80 points), as suggested by the authors of CNN [43]. Two more CNN clustering rounds are introduced to split bulky clusters by increasing the required number of common neighbors (12 and 16). Finally, all clusters with less than 15 points are discarded, retaining only point clouds that span a volume of at least 3.24 Å^3^.

### 3.2. Evaluation

To evaluate the quality of the developed method, the following key questions will be addressed. First, is the T^2^F method able to identify energy hot spots in a cavity where a classical structure-based (SB) approach derives pharmacophore features from the geometry of ligand-target interactions? Second, can additional features (not detected with the SB approach) be highlighted with the T^2^F method and, if yes, how relevant are they for protein binding? To answer these questions, co-crystallized ligands were extracted from PDB files and the T^2^F method was applied on an artificial apo-form of a crystal structure. For each of the five protein classes, one ligand-target complex was randomly picked and a T^2^F model was built after extraction of the ligand and the water molecules (see method section for detailed protocol). Then, this model was aligned and compared to all SB pharmacophore models built for each ligand-protein complex in the same protein family of the set.

#### 3.2.1. Cyclin-Dependent Kinase

**Figure 3:**
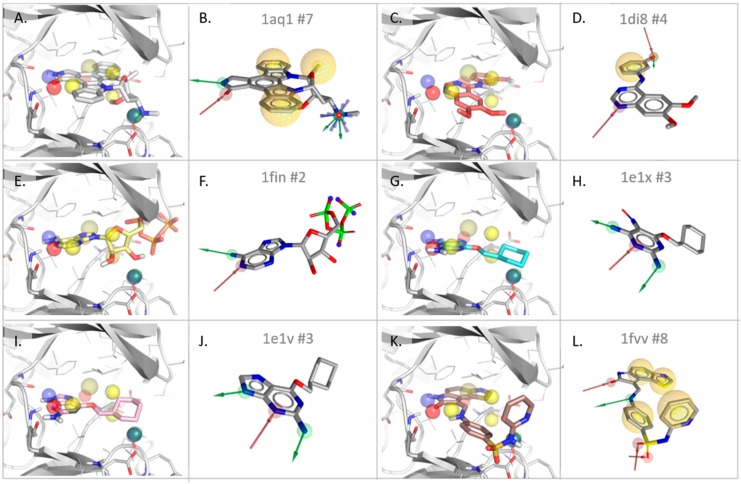
T^2^F model derived from the empty CDK2 cavity (PDB entry 1AQ1) superposed to all CDK2-ligands structures used for the evaluation ((**A**) 1AQ1, (**C**) 1DI8, (**E**) 1FIN, (**G**) 1E1X, (**I**) 1E1V, and (**K**) 1FVV) compared to their corresponding structure-based (SB) pharmacophore models (LigandScout: (**B**) 1AQ1, (**D**) 1DI8, (**F**) 1FIN, (**H**) 1E1X, (**J**) 1E1V, and (**L**) 1FVV). The number of features comprised in the SB models is indicated in the respective subfigures. Color coding in the T^2^F models (first and third column, drawn with PyMol): HBD = blue, HBA = red, H = yellow, and PI = green. Color coding in the SB models (second and fourth column, drawn with LigandScout): HBD = green, HBA = red, H = yellow, and PI = blue.

A T^2^F model was built for Cyclin-dependent kinase 2 (CDK2) using PDB entry 1AQ1 after removing its co-crystalized ligand (STU, an analog of the pan kinase inhibitor, staurosporine). The resulting pharmacophore model includes nine features (Table 4): Four hydrophobic contacts (H), two H-bond donors (HBD), two H-bond acceptors (HBA), and one positive ionizable (PI) feature, as shown in Figure 3. This 3D model was then compared to the SB pharmacophore models derived from the six ligand-kinase complexes of this protein group, including 1AQ1.

Comparing the T^2^F and SB models derived from the same CDK2 structure, 1AQ1, allows a first evaluation of the T^2^F approach (Figure 3A). Seven pharmacophore features are derived from the inhibitor-kinase complex with the SB approach (Figure 3B), of which four are matching T^2^F features with a RMSD of 0.94 Å. The H-bond network (one HBD and one HBA) characteristic of a kinase ATP-binding site (Gln81 and Leu83) in the so-called “hinge region” are identified in both models. Given the importance of this interaction for kinase inhibition [54,55,56], the presence of these two features in the T^2^F model is an essential first validation. In the region of the cavity accommodating the positively charged amine of the ligand (interaction with Gln131 and Asp86), the two models share a second HBD and a PI feature. Interestingly, the hydrophobic features in both models are detected in the same region, but are not perfectly matching (distance of feature center > 2.0 Å). One match is in the backpocket around the aromatic ring (2.1 Å) and another one is at the aromatic ring in the front pocket (2.7 Å). This shift can be explained by the basic principle of SB pharmacophore design, which centers pharmacophore features on chemical groups of the ligand. On the other hand, a method focused on the target identifies the center of the most energetically favorable area for creating hydrophobic contacts, sometimes shifted slightly compared to the ligand coordinates, and sometimes broader than the very position of one particular chemical group of a bound molecule. Furthermore, a large hydrophobic core was identified in the cavity of CDK2, which results in a bulky area of hydrophobic points split up into nearby clusters. While grid points detected as hydrophobic characterize in detail the geometry of a particular hot spot, deriving this complex 3D-volume in a spherical pharmacophore feature is a simplification where some geometrical information can be lost (Figure S1).

Additionally, the T^2^F model of CDK2 identified one HBA that is absent in the SB model of 1AQ1. This feature is identified in a subpocket of CDK2 that is not reached by the co-crystallized ligand. This hot spot highlights a possible interaction with the backbone NH of Asp145, which lies at the entry of a small tunnel in the backpocket filled by several water molecules (e.g., HOH 391 and 392). One H feature not matched by the SB model is in a lipophilic region of the CDK2 pocket, spanned by two aliphatic side chains (Leu134 and Ala144). In total, four hydrophilic features of the SB model are matched in the T^2^F model, the same hydrophobic regions are identified, and one extra HBA feature could be derived, providing additional information on the targeted cavity.

To further evaluate our method, the T^2^F model derived from an emptied CDK2 structure (1AQ1) was compared to SB models derived from other structures of CDK2 co-crystallized with different ligands (Table 4). In the case of the SB pharmacophore of the PDB entry, 1DI8, three H-bond (HB) and one hydrophobic contact (H) were derived from the ligand-kinase complex. Among the three HB features in the SB model (Figure 3D), only the HBA of the hinge region is matched in the T^2^F model (note that the ligand, DTQ500, only forms one hinge HB). Also, the hydrophobic back pocket feature matches one H feature of the T^2^F model (distance 1.84 Å). The two HBs involving the hydroxyl group of DTQ500 in the back pocket were not recorded as a hot spot by the T^2^F approach. This can be explained (i) by the flexibility of this region of the pocket, closed in 1AQ1 and more open in 1DI8 due to ligand binding, and (ii) by the presence of a water molecule (HOH604) interacting with the ligand in the 1DI8 structure. For the PDB entries, 1FIN (Figure 3E,F), 1E1X (Figure 3G,H), and 1E1V (Figure 3I,J), respectively two, three, and three features are detected using an SB approach. These features are HBs formed with the hinge region of CDK2, of which two are matched in the T^2^F model. Finally, the SB model derived from structure 1FVV (holding eight SB features) only matches two features of the T^2^F model: One hydrophobic contact (matched by two SB features) and one HBA (in the hinge region). The binding conformation of the 1FVV ligand (Figure 3K,L) highlights interactions that are different to those of the five other inhibitors of the set. These interactions are located in a region that is very solvent-exposed and not buried enough to be detected using the default settings of the T^2^F method. This explains why two HBA (with the sulfonamide) and one hydrophobic feature (pyridine) were not detected with the T^2^F approach.

In summary, all SB-models shared with the T^2^F model one HB feature in the hinge region, and four SB models shared the two key HB anchor features. However, in other regions of the cavity, four features derived with a T^2^F approach were either too far apart to match or simply absent in the SB models. Therefore, this first step of the evaluation using CDK2 structures demonstrates the ability of the T^2^F tool to not only identify key features for ligand binding, but also new regions that are invisible in an SB approach. Starting from one single structure, the T^2^F model not only detects most features presented by the ensemble of six SB models, but also unveiled novel hot spots that are not covered by any of the six co-crystallized CDK2 inhibitors.

#### 3.2.2. Dihydrofolate Reductase

**Figure 4:**
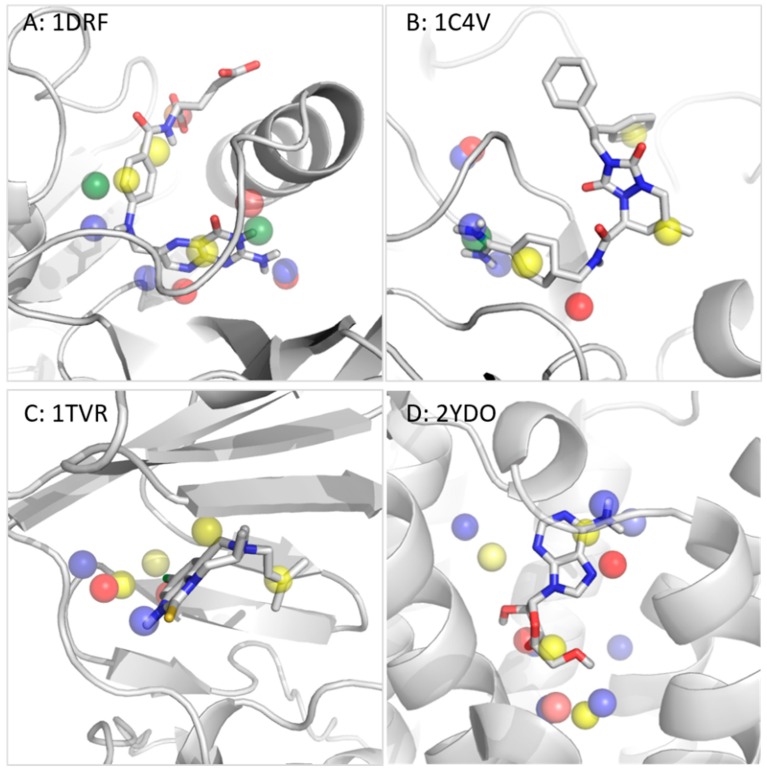
T^2^F models overlaid with the ligand previously extracted from its cavity for the proteins, (**A**) dihydrofolate reductase, (**B**) thrombin, (**C**) reverse transcriptase, and (**D**) adenosine A_2A_ receptor. Color coding in the T^2^F models: HBD = blue, HBA = red, H = yellow, PI = green, and NI = orange.

Dihydrofolate Reductase (DHFR) reduces dihydrofolic acid using cofactor NADPH, which binds in a neighboring pocket to the one targeted with small molecule inhibitors. To derive a target-focused pharmacophore model of this enzyme, the ligand was extracted from PDB entry 1DRF and the T^2^F method was applied. The 13 resulting pharmacophore features are three H, two PI, one NI, four HBA, and three HBD features (Figure 4A and Table S1). The comparison to the SB model derived from 1DRF with the co-crystallized ligand folic acid shows for each of the five SB features a perfect match with one of the T²F features (RMSD = 1.17 Å). This result indicates that the T^2^F approach fully covers the pharmacophoric space detectable with an SB approach for this particular structure, and also identifies additional hot spots in neighboring regions. Interestingly, among these non-matched T^2^F features, two are found on positions very close to a water molecule, bridging an interaction between the ligand and the protein in the co-crystal (HBD with Ser59 and HBA Thr136). Two PI features were detected in the surroundings of the negatively charged side chains of Glu30 and Asp21, respectively. These two features also remained unmatched in the SB model due to the absence of ligand-target interactions with these residues (the water molecule, HOH648, was found to have exactly the same coordinates as the PI feature close to Asp21). Nevertheless, these positions can become important features to consider for DHFR binding. Finally, two more HB (one donor and one acceptor) T^2^F features corresponding to potential interactions with the backbone CO of Val115 and the OH group of Tyr121 remained unmatched. Interestingly, these residues are in the pocket accommodating the dihydropteridin bicycle of the folic acid, in PDB entry 1DRF. Despite the proximity of a nitrogen of the dihydropteridin in the surroundings of these two residues, no interaction was detected with the SB approach. However, we surmise that these interactions can be formed if the dynamics of the system were to be considered. This assumption is also confirmed by the non-matched, but nearby HBD, features detected in other DHFR-ligand complexes of the set (PDB entries 1HFP, IDLR, 1OHK, and 1BOZ). This result indicates that a T^2^F approach to derive a pharmacophore model from a single and static ligand-free structure can detect this type of potential interaction, while the SB approach would require molecular dynamics to access this information [57,58,59].

Comparing the unique T^2^F model (derived from PDB entry 1DRF without a ligand) to the six SB models derived from ligand-DHFR complexes shows that some interactions are shared among this inhibitor class (Table S1). These interactions are represented by the following features, all matched in the T²F model: (a) An HBD with Val8 and Glu30 (shared by four SB models); (b) H with Phe31 and 34, Leu67 and Ile60 (shared by four SB models); and (c) an HBA and NI with Arg70 (shared by four SB models). The six remaining features of the T^2^F model are invisible to the SB approach, highlighting novel anchoring points for further pharmacomodulations. An overlay of the T^2^F model with all ligand-DHFR complexes analyzed in this study is provided in the supporting information (Figure S2).

#### 3.2.3. Thrombin

The third protein chosen to evaluate our method is thrombin, a serine protease for which seven ligand-protein complexes were assembled in the Patel evaluation set [29]. The particularity of the thrombin active site is the geometry of the P1, P2, and P3 pockets, which can recognize and hydrolyze specific peptide sequences. Competitive thrombin inhibitors bind to this region of the enzyme, in particular to the P1 pocket, which is negatively charged due to the presence of Asp189. The PDB entry selected for deriving a T^2^F pharmacophore model is 1C4V. The ligand, IH2370, was extracted from the structure and a target-focused model was built. This model comprises nine features (Figure 4B): Five H-bond features, three H, and one PI features. Two HBD and the PI features are located at the bottom of the P1 subpocket and one H feature is at the entry of this subcavity. One HBA is found in the neighborhood of the catalytic residue, Ser195. Two more H features are detected: One in the subpocket, P2, and one in P3. The last two HB (one donor and one acceptor) detected by the T^2^F approach are in a subpocket hosting two water molecules in the analyzed crystal structure (HOH405 and 408). None of these two HB features are matched by any of the SB models derived from the seven thrombin-ligand complexes in the set, as discussed hereafter.

Out of the nine features in the T^2^F model, six are matched with an RMSD of 0.72 Å in the SB model derived from the same structure, 1C4V with ligand IH2370 (also comprising nine features, see Table S2). Features in pockets P1, P2, and P3 are all identified with an SB and T^2^F approach. However, the T^2^F method could not detect two H-bond features derived from the ligand-thrombin complex involving Gly216 found in the SB models. The reason for this is that this residue is located at the edge between the subpockets, P2 and P3, in a non-buried region that is solvent exposed in absence of a ligand. One of the four H features found in the SB model was also absent from the T^2^F model for the same reason. Besides the two HB identified in an allosteric pocket, the singularity of the T^2^F model is that one HBA was identified in the region of the backbone NHs of Ser195 and Gly193.

A very similar result comes out of the detailed analysis of the six other SB models derived from the remaining thrombin-ligand complexes in the set (the T^2^F model superposed to all ligand-thrombin complexes is shown in Figure S3). On the one hand, all crystallized inhibitors also form between one and two H-bonds at the edge between the P2-P3 subpockets (Gly216) that are not detected with the T^2^F approach due to the buriedness criterion (apart from PDB entry 1D4P, where no SB hydrogen bond features are found in this region for this particular ligand-thrombin complex). On the other hand, the pharmacophore features, PI and HBD, of the electrophilic P1 subpocket are detected in all structures by both methods (except for PDB entry 1D6W for which only two HBDs are derived due to the neutral charged assigned to the bound aminoimidazol group). Similarly, the H feature of the lipophilic P3 subpocket is found in all SB models, while the H feature of the P2 pocket is only detected for PDB entries, IC4V and 1FPC, leaving this key feature invisible to an SB approach in other crystal structures.

#### 3.2.4. Reverse Transcriptase

HIV Reverse Transcriptase (RT) is a DNA polymerase extensively studied for the development of anti-retrovirus therapy. The binding site of this protein is very lipophilic, which is why some parameters were adjusted to derive the T^2^F-Pharm model of this protein (see methods section). Scanning empty PDB entry 1TVR with the T^2^F tool highlighted eight hot spots (Figure 4C): Four H features, two HBD, and two HBA features. Among them, four are matched by the SB model derived from the same structure with a ligand (three H and one HBD features), with an RMSD of 0.76 Å. Two SB features are not detected as hot spots in the T^2^F approach. The first one is an HBA with the NH backbone of Lys101, which is detected in the SB model only if the ligand is minimized in the cavity. The second non-matched feature of the SB model is one of two H features derived by LigandScout from the short aliphatic chain flanking the core of the inhibitor. In this area, the T²F-Pharm clustering procedure returns only one H hot spot.

Among the eight features detected with a T^2^F approach, seven are matched by at least two SB-models from all 10 RT complexes (Table S3). Interestingly, not a single T^2^F feature is matched by all SB models, indicating that no model fully covers the spectrum of possible interactions to interact with RT. In fact, no SB-derived feature is shared by all SB models, illustrating the diversity of the pharmacophoric space for this cavity. Finally, one T^2^F-derived feature that is absent in all SB models is an HBA hotspot identified between the OH group of Tyr318 and the backbone NH of His 235. These results illustrate the quality of this model derived from a unique empty cavity in contrast with the ten SB-model (the T^2^F model superposed to all ligand-RT complexes is shown in Figure S4).

#### 3.2.5. Adenosine A_2A_ Receptor

G-protein coupled receptors (GPCRs) are an important group of proteins for which small molecule binders represent a large proportion of the drugs on the market [60]. Among them, adenosine receptors have become central therapeutic targets for the treatment of various pathologies (cardiovascular, renal, and nervous systems, as well as endocrine and pulmonary disorders) for more than a decade [61]. To further evaluate our method, we derived a T^2^F pharmacophore model from the A_2A_ receptor structure after extracting the co-crystalized adenosine (PDB entry 2YDO) and compared it to SB models derived from crystal structures with (a) adenosine, (b) the synthetic agonist, NECA, and (c) the inverse agonist, ZM241385 [62] (Table 2).

With parameters adapted to the large and hydrophilic cavity of A_2A_, a T^2^F model, comprising 15 features, was derived, including seven HBD, four HBA, and four H features (Table S4). Out of the seven features detected with an SB approach with the bound adenosine, six are matched by the T^2^F model (RMSD = 1.1 Å), as shown in Figure 4D. The non-matched feature in the SB model is a second HBD between a hydroxyl group of the ribose of the ligand and His278. The T^2^F algorithm derives in this region one single hotspot and centers this cluster on the neighboring HBD of the SB model (adjacent to the OH group interacting with the same His278 residue as well as with Ser277). The comparison of the T^2^F model extracted from the empty PDB entry 2YDO with the structure-based model derived from the A_2A_ receptor in the complex with the agonist NECA (PDB entry 2YDV) shows similarly good results. Here, eight out of nine features are matched (RMSD 1.2 Å) and the last HBD is not matched by the T^2^F model for exactly the same reason as discussed in the previous example. Finally, an SB model derived from a slightly different receptor conformation (PDB entry 3EML) hosting an A_2A_-inverse agonist was compared to the T^2^F model. In this case, three out of five features of the SB model derived from the interactions detected with the ligand, ZM241385 (PDB entry 3EML), are matched by the T^2^F model. The two non-matched SB features are hydrophobic contacts (H). The first one is 2.36 Å apart from the closest H features of the T^2^F model, thus, almost matching the 2.00 Å distance cut-off. The second unmet SB feature is detected on the phenol ring at the pocket entrance, which is outside of the grid built from the 2YDO pocket in the reference T^2^F model. Interestingly, eight features detected by T^2^F-Pharm in the large GPCR binding site are invisible to the SB approach. Note that water molecules were not included in the T^2^F calculation, while the adenine ring in the x-ray structure is stabilized through a water network. Three of the unmet T^2^F features are overlapping with the position of these water molecules. Furthermore, one unfulfilled hydrophobic feature partially overlaps with the aromatic adenine ring moiety of all three ligand structures. The remaining features are located in sub-pockets not reached by these three ligands, delivering information of additional potential hotspots for ligand binding.

#### 3.2.6. Sensitivity to Conformational Changes

The presented evaluation was conducted for five different protein classes by choosing randomly one structure to derive a reference T^2^F pharmacophore. Thus, it appears important to analyze the sensitivity of the method to conformational changes of the protein structure selected for T^2^F modeling. Therefore, one T^2^F-Pharm model was derived from each of the six CDK2 kinase structures considered in this study (after removing their co-crystallized ligands) and the number of matching features was measured for each pair of T^2^F-Pharm models using the 2.00 Å distance cut-off. While some pairs exhibit high similarity (e.g., pairs: 1E1V-1E1X: 100%, 1FIN-1FVV: 80%, or 1AQ1-1FVV: 67% features matched), some show low similarity (e.g., pairs: 1E1V-1FIN: 20%), indicating a sensitivity of the method to the protein conformation under investigation. After a superposition on the Cα atoms of all structures, an analysis of the RMSD of all atoms (including side chains) of the CDK2 binding site (residues within 6 Å around the co-crystalized ligand STU) shows deviations between 2.1 Å for the 1E1V-1E1X pair to 14.9 Å for the same residues for the 1E1V-1FIN pair. Thus, on the one hand, the ability of the method to distinguish between enzyme conformations can be considered a strength, while on the other hand, a too high sensitivity regarding minor side chain movements is not desirable.

## 4. Conclusions

The landscape of available tools for translating protein surfaces into hot spots for optimal binding in the absence of ligand information is extremely limited. For that reason, we developed the T²F method for target-focused pharmacophore modeling, an innovative approach based on an elaborated cavity annotation method, combined with hot spot filtering and the advanced common nearest neighbor (CNN) clustering method [41]. The targeted biomolecular surface can be defined manually by the user, derived from the center of mass of a co-crystallized ligand, or by using a cavity detection tool.

An evaluation of our method was conducted with five structurally different enzymes, demonstrating its ability to identify key hot spots for binding to a biomolecular cavity. The presented work shows that most key features derived from the geometry of multiple ligand-target complexes with an SB approach can also be detected by scanning the energy landscape of a single empty protein structure. The five presented cases show how a T^2^F model derived from one unique structure can highlight a set of hot spots that an SB approach only accesses if multiple ligand-protein structures are available. Moreover, we showed that the T^2^F tool can detect hot spots that remained invisible to a classical SB approach.

In some cases, particular features identified with an SB method were not observed using default parameters. To overcome this issue, an acute knowledge of the studied structure is required to fine tune the parameters, in particular the buriedness cut-off PSP (e.g., thrombin case) or hydrophobicity cut-off (e.g., reverse transcriptase case). Also, opting for spherical pharmacophore features results in a loss of geometrical details on the studied cavity. However, this simplification is indispensable as most 3D modeling and pharmacophore screening software can only process pharmacophore models with features represented as spheres. Nevertheless, the size of the sphere representing a T^2^F feature is proportional to the amount of grid points contained in the derived cluster, conserving key spatial information about the cavity that is absent in an SB approach.

Finally, an analysis of the variability of the derived pharmacophore features with respect to the selected reference structure showed that the method is sensitive to changes in side chain orientation observed in closely related crystal structures. To have a better control of the impact of side chain flexibility, we are currently working on incorporating pocket flexibility, e.g., using structural ensembles or molecular dynamic trajectories, to derive a dynamic T^2^F pharmacophore.

In conclusion, this preliminary evaluation demonstrates a promising robustness of the T^2^F method to derive important hot spots from empty biomolecules. This information can be particularly useful to analyze underexplored protein cavities or targets with no known inhibitors. Additionally, T^2^F-Pharm outputs pharmacophore models that can be used for docking and virtual screening, as well as in the investigation of protein allosteric pockets or protein-protein interactions. Therefore, the presented approach not only represents a novel tool for drug discovery, but also a valuable instrument to investigate protein surfaces in the absence of known binding partners. We believe that this simple and straightforward tool can deliver meaningful pharmacophore models for unexplored proteins, but is also complementary to the SB approach, e.g., to identify potential interactions to a target in the context of a lead expansion program.

## Figures and Tables

**Table 1:** List of the discussed energy-based methods relying on geometry or a grid for target-focused pharmacophore modeling.

Method	Cavity Definition	Approach	Clustering Method	Evaluation	Year	Refs.
*Ph4Dock*	cavity detection (Delaunay triangulation/α spheres)	electrostatic interactions (MMFF94 [26]) of charged dummy atoms	single-linkage	CCDC/Astex valida-tion set [27] ^d^	2004	[18]
*Pocket V2*	grid box around ligand (or user-defined pocket residues)	grid (Score) [28]	unclear clustering method ^a^	CDK2, HIV1-PR, ER, 17b-HSD	2006	[19]
*FLAP* + *BioGPS*	grid box around ligand or FLAPsite detection	grid (GRID software) [17]	region-based energy minima	Patel set [29], DUD [30] ^d^	2007	[23,31]
*Tintori* et al.	grid box around binding site	grid (GRID software) [17]	no clustering ^b^ (GRID minima + interpolation)	TrxR (MTB), HIV1 IN, HIV-1 RT dimer	2008	[21]
*Hydro-Pharm*	grid box around ligand (3 Å)	grid (ChemScore [25]) + MD-based hydration site feature reduction ^c^	k-means	HIV1-PR, DHFR, FXa	2012	[22]
*PharmDock*	grid box around bound ligand (3 Å)	grid (ChemScore [25])	k-means	PDB bind, DUD [30] ^d^	2014	[24,32]
*T^2^F-Pharm*	grid box around ligand or user-defined center (& cavity point reduction)	grid (AutoDock) [33]	CNN [34]	Patel set [29] + A_2A_ receptor	2018	This paper

^a^ Exact clustering method not specified in publication. ^b^ No clustering method used, points are reduced using the Minim and Filmap programs implemented in the GRID package, collecting all points within a certain energy threshold value and interpolating the closest ones. ^c^ Hydro-Pharms adds a second step to further restrict the pharmacophore points by calculating an overlap between the grid-based pharmacophores and molecular dynamics (MD)-derived hydration sites. ^d^ Evaluation focused on enrichment in docking/virtual screening, rather than evaluation of pharmacophoric features.

**Table 2:** Evaluation data set.

Group	Reference Structure (Ligand)	Water	Others Structures
Cyclin-dependent kinase 2 (CDK2)	1AQ1 (STU)	-	1E1X, 1FVV, 1DI8, 1E1V, 1FIN
Dihydrofolate reductase (DHFR)	1DRF (FOL)	-	1BOZ, 1DLR, 2DHF, 1OHK, 1HFP
Thrombin	1C4V (IH2)	HOH 404, 408, 410 and 477	1D4P, 1D6W, 1D9I, 1DWD, 1TOM, 1FPC
HIV-reverse transcriptase (RT)	1TVR (TB9)	-	1DTT, 1EP4, 1FK9, 1RT1, 1RT3, 1VRU, 1RT5, 1KLM, 1BMQ
A_2A_ receptor	2DYO (ADN)	-	2YDV, 3EML

**Table 3:** Default parameters used for T^2^F pharmacophore elucidation.

Grid box	Center	Ligand CoM * or center coordinates
	Size of the edge of the cubic box	16 Å
	Distance between two grid points	0.6 Å
Cavity	Occupancy	0.6 kcal/mol
	Buriedness (PSP)	4
Feature type	Hydrophilic radius	3 Å
Type specific energy cut-off **	Hydrophobic (H)	−0.4 kcal/mol (−0.6)
	H-bond donor (HBD)	−0.35 kcal/mol (−0.3)
	H-bond acceptor (HBA)	−0.6 kcal/mol (−0.5)
	Negative/Positive ionizable (NI/PI)	±1.0 kcal/mol
Clustering	Neighbor distance cut-off	1.21 Å
	Number of common neighbors	6 (12, 16) ***
	Min. number of points per cluster	15

* CoM = center of mass. ** Numbers in parentheses are parameter values used for the hydrophobic pocket of reverse transcriptase (PDB entry 1TVR). *** Numbers in parentheses are parameter values used for splitting larger clusters in second and third rounds.

**Table 4:** Feature overlap for the T^2^F model derived from the empty CDK2 cavity (empty 1AQ1).

Type	Dist **	Freq ***	1AQ1	1DI8	1FIN	1E1V	1E1X	1FVV
#match *	-	-	4/7	2/4	2/2	2/3	2/3	3/8
rmsd ****			0.94	1.59	0.50	0.69	0.96	1.18
HBD	0.56	4	X		X	X	X	
HBA	0.45	6	X	X	X	X	X	X
HBD	1.00	1	X					
PI	1.17	1	X					
H	1.39	2	X (2.1 Å)	X				2 * X
H			X (2.7 Å)	Slightly shifted front pocket H feature				
H	Surrounding of Leu134 and Ala144							
H	Not detected in SB models							
HBA	HBA towards back pocket water channel (ASP 145, backbone NH)							

* Match: Number of matches between T²F and SB pharmacophore features in relation to number of SB pharmacophores features in the respective SB model. ** dist: Minimum distance (in Å) of the respective matching features from the different SB models. *** freq: Number of protein structures that exhibit this T²F -SB feature match. Notes in light grey are comments referring to features close to a match but more distant than 2 Å. **** RMSD describes the root-mean-square deviation (RMSD) in Å of the matching T^2^F and structure-based (SB) features.

## Supplementary Materials

Supplementary data associated with this article can be accessed with the online version.
