# Supplementary material for: Truly Target-Focused Pharmacophore Modeling: A Novel Tool for Mapping Intermolecular Surfaces
Source: Molecules. 2018 Aug 6;23(8):1959. doi: 10.3390/molecules23081959 (PMC6222449; doi:10.3390/molecules23081959)
Supplement: Supplementary file 1 [file molecules-23-01959-s001.docx]

**Truly target-focused pharmacophore modeling:**

**a novel tool for mapping intermolecular surfaces**

Jérémie Mortier, Pratik Dhakal, Andrea Volkamer*

Charité – Universitätsmedizin Berlin, Institute of Physiology, In-silico Toxicology Group, Virchowweg 6, 10117 Berlin, Germany

* Correspondence: [andrea.volkamer@charite.de](mailto:andrea.volkamer@charite.de)

**Supporting material**


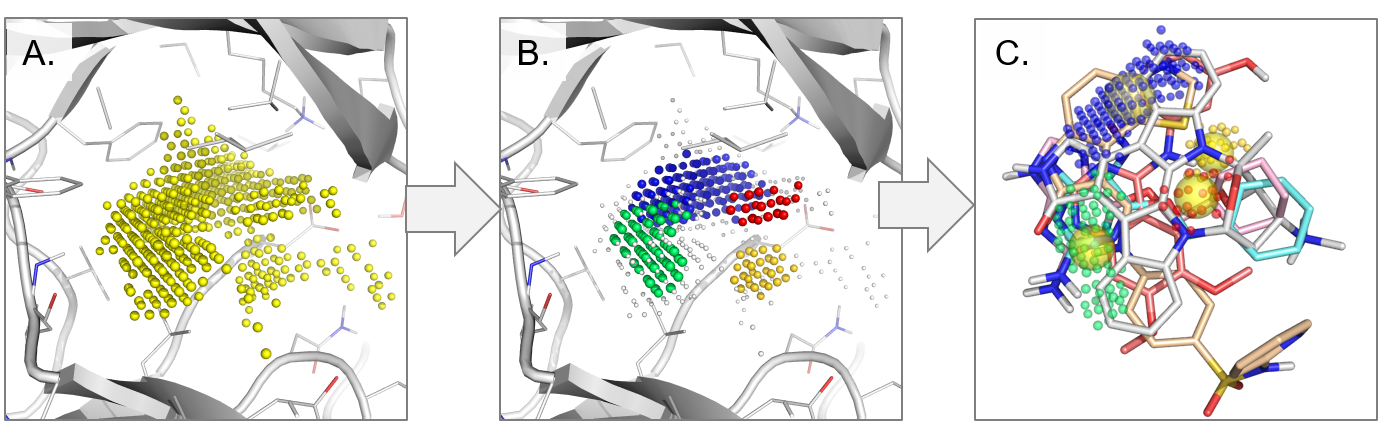


**Figure S1.** Clustering procedure and resulting pharmacophore features exemplified for grid points identified as hydrophobic in CDK2 (empty 1AQ1). A: All hydrophobic grid points remaining after energy cut-off. B: CNN clusters (green, blue, red yellow) and noise points (gray). C: Overlap of grid points and pharmacophore features (yellow spheres) with all ligands from reference set.


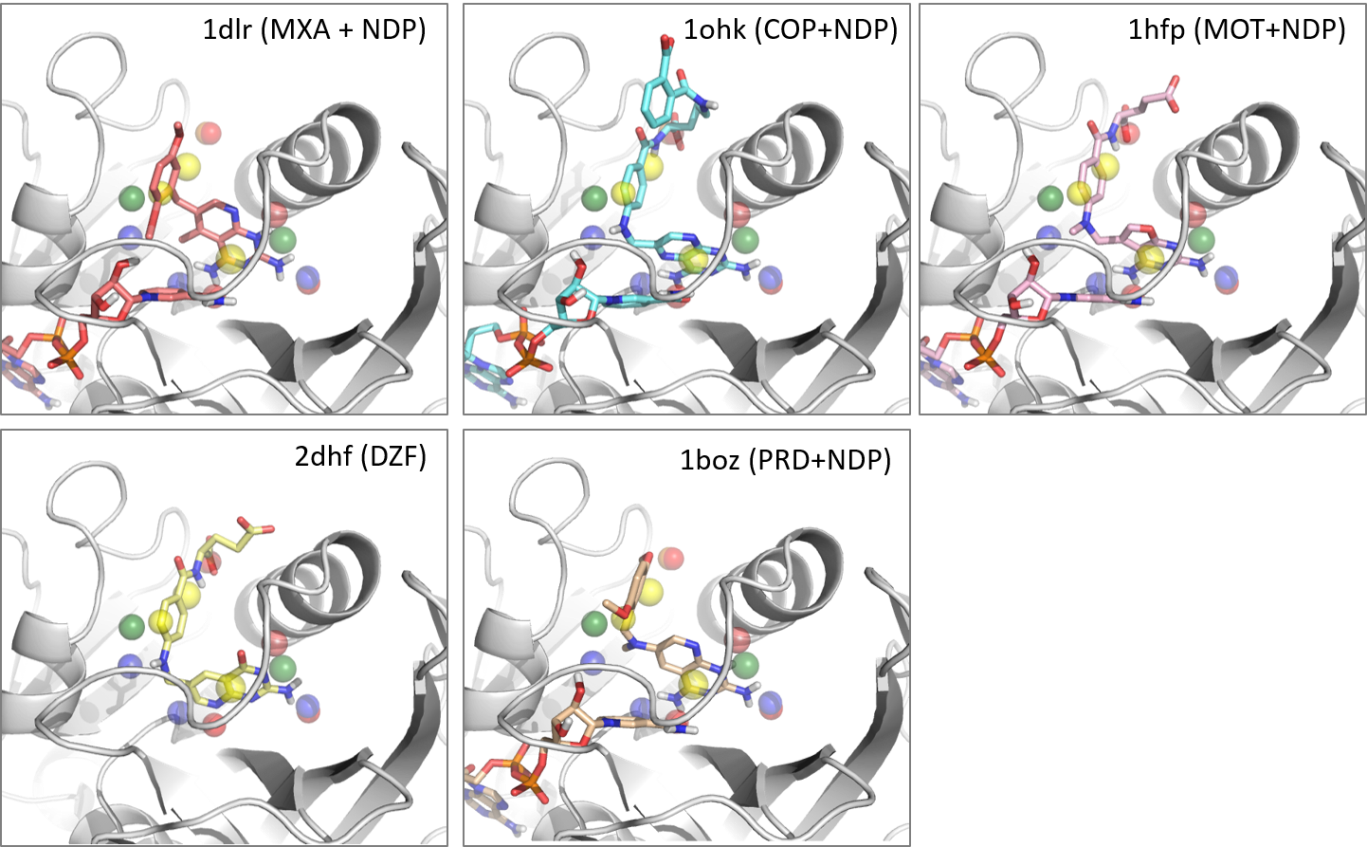


**Figure S2.** T^2^F model derived from the DHFR cavity (empty 1DRF) superposed to all ligands from the five reference DHFR-complexes (including cofactors) used for the evaluation: 1DLR, 1OHK, 1HFP, 2DHF, and 1BOZ (ligand 3-letter code in parantheses). Color coding in the T^2^F models: HBD = blue, HBA = red, H = yellow, PI = green, NI = orange.

**Table S1.** Feature overlap for T^2^F model derived from the DHFR cavity (empty 1DRF).

| type | dist^**^ | freq^***^ | | 1drf | | | 1dlr | 1hfp | | 1ohk | | | 2dhf | 1boz | |  |
| --- | --- | --- | --- | --- | --- | --- | --- | --- | --- | --- | --- | --- | --- | --- | --- | --- |
| #matches / #SB features* | | | | 5/5 | | | 1/4 | 4/6 | | 5/8 | | | 5/5 | 1/3 | |  |
| match RMSD [Å] **^****^** | | | | 1.17 | | | 1.3 | 1.17 | | 1.18 | | | 1.24 | 1.82 | |  |
| H | 1.22 | 4 | | X | | | X |  | | X | | | X | X (2.4 Å) | |  |
| H | 1.74 | 1 | |  | | |  | X | |  | | |  |  | |  |
| HBD | 1.65 | 4 | | X | | |  | X (2.0 Å) | | X | | | X | X | |  |
| HBA | 0.42 | 4 | | 2*X | | |  | 2*X | | 2*X | | | 2*X |  | |  |
| NI | 0.53 | 4 | | X | | |  | X | | X | | | X |  | |  |
| HBA | Not detected in SB models | | | | | | | | | | | | | | |  |
| HBA | Overlap with water HOH656 close to Thr136 | | | | | | | | | | | | | | |  |
| HBA | Not detected in SB models | | | | | | | | | | | | | | |  |
| PI | Overlap with HOH648 close to Asp21 | | | | | | | | | | | | | | |  |
| PI | Not detected in SB models (close to Glu30) | | | | | | | | | | | | | | |  |
| H | Not detected in SB models | | | | | | | | | | | | | | |  |
| HBD |  | |  | |  | X (2.5 Å) | | | X (2.4 Å) | | X (2.4 Å) |  | | | X (2.5 Å) |  |
| HBD | Not detected in SB models (close to Ser59) | | | | | | | | | | | | | | |  |

^*^match: Number of matches ìn T²F / SB pharmacophore features in relation to number of SB pharmacophores features in the respective SB model. ^**^dist: Minimum distance (in Å) of the respective matching features from the different SB models. ^***^freq: Number of protein structures that exhibit this T²F-SB feature match. Notes in light grey are comments referring to features close to a match but more distant than 2.00 Å. **^****^**RMSD describes the root-mean-square-deviation RMSD in Å of the matching T^2^F and structure-based (SB) features.


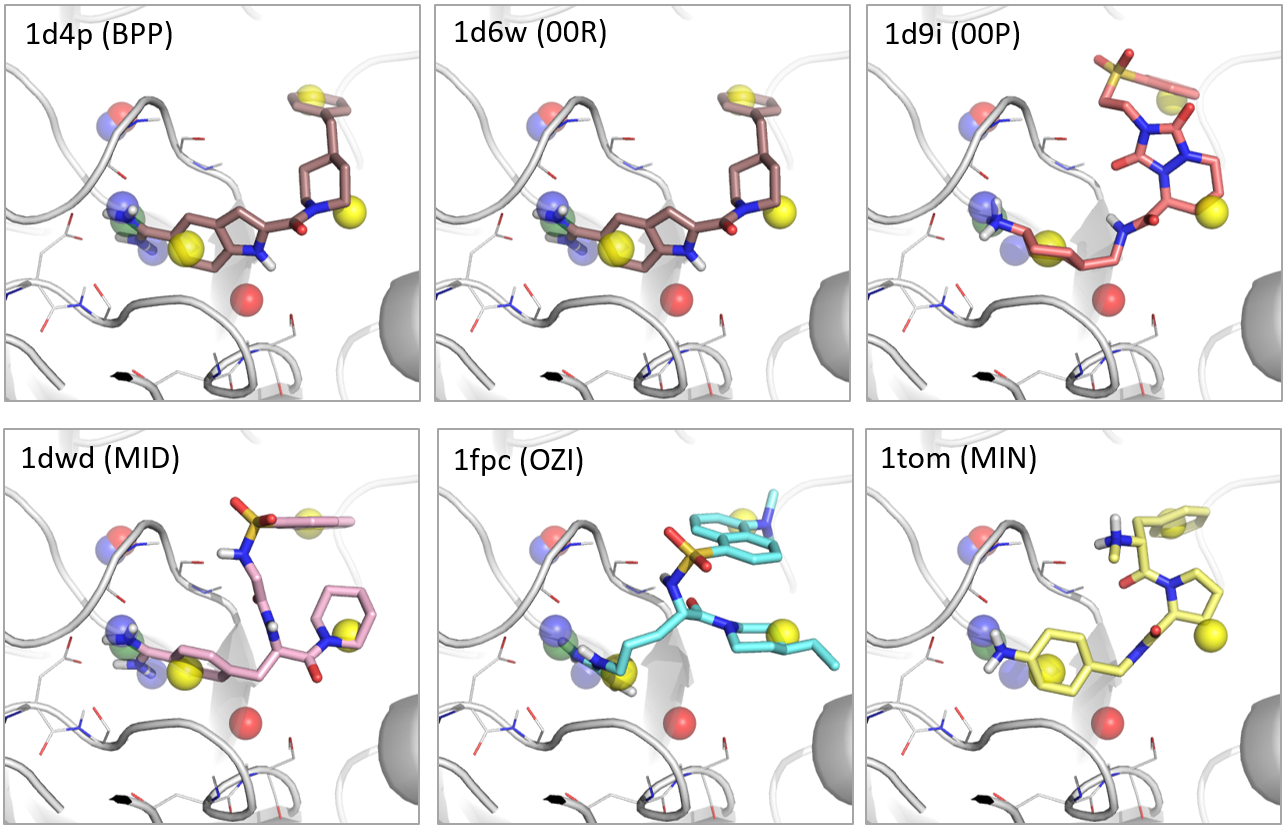


**Figure S3.** T^2^F model derived from the thrombin cavity (empty 1C4V) superposed to all ligands from the six thrombin-complexes used for the evaluation: 1D4P, 1D6W, 1D9I, 1DWD, 1FPC, 1TOM (ligand 3-letter code in parentheses). Color coding in the T^2^F models: HBD = blue, HBA = red, H = yellow, PI = green, NI = orange.

**Table S2.** Feature overlap for T^2^F model derived from the thrombin cavity (empty 1C4V).

| type | dist^**^ | freq^***^ | 1cv4 | 1d4p | 1d6w | 1d9i | 1dwd | 1fpc | 1tom |
| --- | --- | --- | --- | --- | --- | --- | --- | --- | --- |
| #match / #SB features* | | | 6/9 | 5/5 | 3/5 | 3/5 | 5/8 | 5/10 | 3/6 |
| match RMSD [Å] **^****^** | | | 0.72 | 0.76 | 1.39 | 1.31 | 0.88 | 1.67 | 1.22 |
| HBD | 0.39 | 7 | X | X | 2*X | X | X | X | X |
| HBD | 1.23 | 4 | X | X |  |  | X | 2*X |  |
| PI | 0.47 | 6 | X | X |  | X | X | X | X |
| H | 0.68 | 7 | X | X | X | X | X | X | X |
| H | 0.38 | 1 | X |  |  |  |  | X (2.6Å) |  |
| H | 0.61 | 3 | X | X |  |  | X |  |  |
| HBA | Potential HBA anchor position (around Ser195) | | | | | | | | |
| HBA | Features in small subpocket filled with a water molecule | | | | | | | | |
| HBD |  |  |  |  |  |  |  |  |  |

^*^match: Number of matches between T²F and SB pharmacophore features in relation to number of SB pharmacophores features in the respective SB model. ^**^dist: Minimum distance (in Å) of the respective matching features from the different SB models. ^***^freq: Number of protein structures that exhibit this T²F-SB feature match. Notes in light grey are comments referring to features close to a match but more distant than 2 Å. **^****^**RMSD describes the root-mean-square-deviation RMSD in Å of the matching T^2^F and structure-based (SB) features.


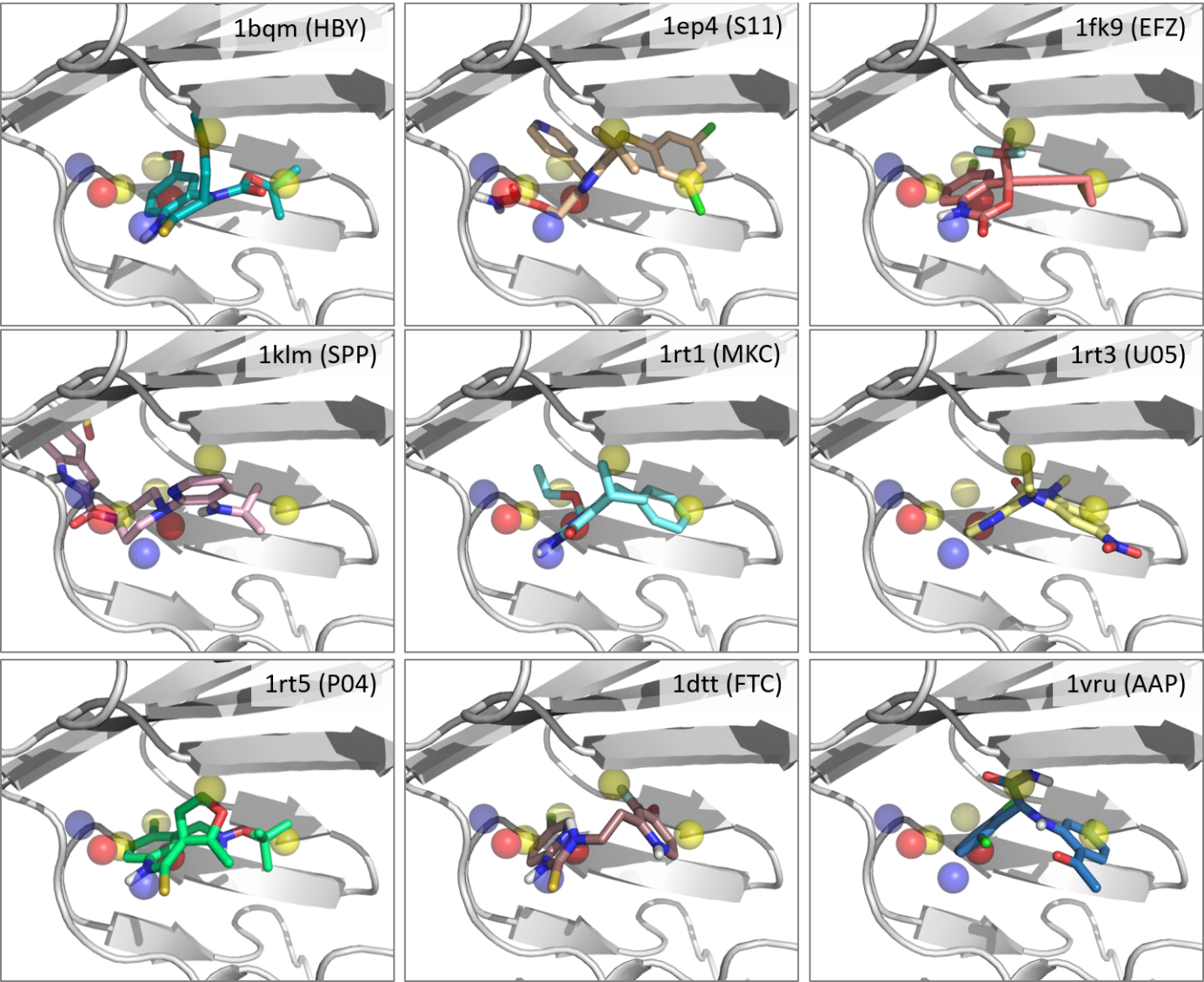


**Figure S4.** T^2^F model derived from the reverse transcriptase cavity (empty 1TVR) superposed to all RT-ligand complexes used for the evaluation. Color coding in the T^2^F models: HBD = blue, HBA = red, H = yellow, PI = green, NI = orange.

**Table S3.** Feature overlap for T^2^F model derived from the reverse transcriptase cavity (empty 1TVR).

| type | dist^**^ | freq | 1tvr | 1bqm | 1ep4 | 1fk9 | 1klm | 1rt1 | 1rt3 | 1rt5 | 1dtt | 1vru |
| --- | --- | --- | --- | --- | --- | --- | --- | --- | --- | --- | --- | --- |
| #match / #SB features* | | | 4/6 | 4/5 | 5/8 | 5/7 | 4/5 | 4/4 | 2/3 | 5/7 | 3/7 | 2/6 |
| match RMSD [Å] **^****^** | | | 0.76 | 1.13 | 1.25 | 1.00 | 1.55 | 1.24 | 0.77 | 1.04 | 0.73 | 1.37 |
| H | 0.82 | 8 | X | 2*X | X | X | X | X | X | X |  |  |
| H | 0.31 | 9 | X | X | X | X | X | X | X | X |  | 2*X |
| H | 0.18 | 6 | X |  | X | X |  | X |  | X | X |  |
| HBD | 0.62 | 6 | X | X |  | X |  | X |  | X | X |  |
| H | 1.09 | 3 |  |  |  | X |  |  |  | X | X |  |
| HBD | 1.57 | 2 |  |  | X |  | X |  |  |  |  |  |
| HBA | 0.59 | 2 |  |  | X |  | X |  |  |  |  |  |
| HBA | Potential HBA anchor position (close to Tyr318 and His235) | | | | | | | | | | | |

^*^match: Number of matches between T²F and SB pharmacophore features in relation to number of SB pharmacophores features in the respective SB model. ^**^dist: Minimum distance (in Å) of the respective matching features from the different SB models. ^***^freq: Number of protein structures that exhibit this T²F-SB feature match. Notes in light grey are comments referring to features close to a match but more distant than 2 Å. **^****^**RMSD describes the root-mean-square-deviation RMSD in Å of the matching T^2^F and structure-based (SB) features.

**Table S4.** Feature overlap for the T^2^F model derived from the adenosine A_2A_ receptor cavity (empty 2YDO).

| type | dist^**^ | freq | 2ydo | 2ydv | 3eml |
| --- | --- | --- | --- | --- | --- |
| #match / #SB features* | | | 6/7 | 8/9 | 3/5 |
| match RMSD [Å] **^****^** | | | 1.05 | 1.22 | 1.30 |
| HBD | 0.76 | 4 | 2*X | 2*X |  |
| HBD | 1.60 | 1 |  | X |  |
| HBD | 1.26 | 6 | 2*X | 2*X | 2*X |
| HBA | 1.25 | 3 | X | X | X |
| HBA | 0.41 | 2 | X | X |  |
| H | 0.96 | 1 |  | X |  |
| H | 2.37 | 1 |  |  | X |
| H |  | Overlap with the adenine ring | | | |
| H |  |  |  |  |  |
| HBD |  | Overlap with water molecule | | | |
| HBD |  | Overlap with water molecule | | | |
| HBD |  |  |  |  |  |
| HBD |  |  |  |  |  |
| HBA |  | Overlap with water molecule | | | |
| HBA |  |  |  |  |  |

^*^match: Number of matches between T²F and SB pharmacophore features in relation to number of SB pharmacophores features in the respective SB model. ^**^dist: Minimum distance (in Å) of the respective matching features from the different SB models. ^***^freq: Number of protein structures that exhibit this T²F-SB feature match. Notes in light grey are comments referring to features close to a match but more distant than 2 Å. **^****^**RMSD describes the root-mean-square-deviation RMSD in Å of the matching T^2^F and structure-based (SB) features.
